# Egg Quality from Siciliana and Livorno Italian Autochthonous Chicken Breeds Reared in Organic System

**DOI:** 10.3390/ani10050864

**Published:** 2020-05-16

**Authors:** Ambra R. Di Rosa, Biagina Chiofalo, Vittorio Lo Presti, Vincenzo Chiofalo, Luigi Liotta

**Affiliations:** 1 Department of Veterinary Sciences, University of Messina, 98168 Messina, Italy; biagina.chiofalo@unime.it (B.C.); vittorio.lopresti@unime.it (V.L.P.); luigi.liotta@unime.it (L.L.); 2 Consortium Research of Meat and Agrifood, 98168 Messina, Italy; vincenzo.chiofalo@unime.it; 3 Department of Chemical, Biological, Pharmaceutical and Environmental Sciences, University of Messina, 98166 Messina, Italy

**Keywords:** Siciliana chicken breed, Livorno chicken breed, eggs quality, biodiversity safeguard

## Abstract

**Simple Summary:**

Nowadays, biodiversity is becoming increasingly important every day, both for its interest in safeguarding biodiversity and because the reduction of genetic variability leads animals to a poorer response to ever faster and more unexpected environmental and climatic variations. Moreover, while nutritional differences among foods obtained from the most widely used livestock species have been relatively well documented, less attention has been paid to foods obtained from different breeds within species. According to the Food and Agriculture Organization FAO objective for native breed safeguarding, the aim of this study was to present a first contribution on the egg quality of endangered the Siciliana chicken breed comparing these results with those of Livorno pure breed, reared in the same organic condition system to obtain useful data for future conservation programs. The results of our research showed similar values for the physical–chemical characteristics, fatty acid profile, and nutritional indices of Siciliana and Livorno eggs, highlighting several valuable quality traits of eggs from these breeds which might be taken into account for the conservation and the exploitation of this low today utilized Italian chicken. Therefore, the results of our research must be considered as an original set of knowledge useful to encourage farmers rearing autochthonous breeds, particularly suitable for organic systems.

**Abstract:**

In poultry production, the intensive use of high-performing hybrid animals led to loss of genetic variability and a consequent lower response to climatic change and disease. Poultry biodiversity is seriously threatened, and its safeguard is a strong objective in developed countries. According to the FAO, which emphasized the importance of native breeds for its country of origin, the aim of this study was to present the first contribution on eggs quality for endangered the Siciliana chicken breed and deepen knowledge on the local Livorno breed. At 20 weeks of age, 108 laying hens (54 Siciliana breed and 54 Livorno breed) were divided into six homogeneous groups of 18 hens each and reared according to requirements imposed by the EC Regulation 889/08 for organic production. The production cycle was controlled over one year, and egg production was recorded daily by group. Eggs were collected, weighted, and measured. Physico-chemical parameter and fatty acids profile were analyzed and nutritional indexes calculated. The statistical model included the effects of breed (Siciliana, Livorno). Egg production was 190 egg/head for Siciliana and 180 for Livorno group. The results showed similar values for Siciliana and Livorno egg quality, highlighting several valuable quality traits from these breeds which might be taken into account for conservation programs.

## 1. Introduction

In developed countries, and among them Italy, egg production is obtained from hybrid varieties of hens, selected for improved performance and bred in controlled conditions. However, consumers, in more affluent societies have for some time also begun to consider, when deciding on their diet, a host of broader concerns relating to themes such as the environment, biodiversity, climate change, animal welfare, and the social condition of the people producing the food. So, nowadays, biodiversity has become more important, both for its interest in safeguarding biodiversity and because the reduction of genetic variability leads to a worse and less effective response to ever faster and more unexpected environmental and climatic variations. Furthermore, the breeding of commercial hybrids of hens reduces the livestock activity in the areas with marginal economic potentialities. 

Moreover, little attention has been given to foods obtained from specific breeds. Recent years have, however, shown growing interest in food biodiversity, defined as “food identified at the taxonomic level below the species level, and underutilized or wild species” [1]. The ever-growing demand for food and the consequent competition for land and resources, in addition to the continuous climatic changes, pose strong concerns for the sustainability of animal production systems and their impact on the environment [2].

In order to assure a good welfare status, the EC Regulations and the final recommendation of the Network for Animal Health and Welfare in Organic Agriculture [3,4] suggest to utilize local breeds [5,6,7,8] and slow-growing birds (daily weight gain < 35 g) [9] for their higher adaptability to poorer environment, for to counter losses of the biodiversity [10].

In Italy, the number of the endangered autochthonous chicken breeds has dramatically increased leading to the current critical situation. Zanon and Sabbioni [11] reported that approximately 61% of the Italian autochthonous avian breeds must be considered as extinct, 13% threatened, 17% scarcely diffuse, and 9% diffused [12]. The introduction of commercial species, selected to maximize the yields and the creation of the specialized crossbreeds for several productions, led to the significant reduction in genetic diversity that is ongoing globally and many local chicken breeds in Italy became extinct, as has happened in the other countries of the world [13,14].

Despite all these limitations, today 13 local chicken breeds still exist and are protected as part of various conservation programs in various Italian regions [15].

The Siciliana is an ancient Italian breed of chicken from Sicily island. The breed had declined, and in the 1980s it was almost extinct. In 1986, some examples were shown at Forlì and interest in the breed was renewed [16]. In 2004, the Siciliana was included in the official standard of the Federazione Italiana Associazioni Avicole, the federation of the Italian poultry associations which is the national authority governing poultry breeding in Italy [17,18]. With Ministerial Decree No. 1936 dated 01.10.2014, the Ministry of Agricultural, Food and Forestry Policies (MIPAAF) [19] established the “Registry of the Autochthonous Poultry Breeds” and approved the chicken breed Standards with 20 local chicken breeds. The breed numbers remain low. A study published in 2007 used a figure of approximately 1000 for the total breeding stock, of which approximately 250 were cocks [20].

The Livorno, or Leghorn, is an Italian breed of chicken known all over the world. The breed derives from crosses of chickens reared in the central Italy countryside, and took its name from the Livorno city. It is a light and lively breed, an excellent white egg layer, but rarely go broody. The mean production can reach two-hundred and eighty eggs per year; the feed-to-egg conversion rate is excellent [21]. For this reasons, many foreign countries have made a careful selection starting from the original Italian chicken, so today there are also the American Livorno (Leghorn call), the German Livorno (called Italiener), and the English Livorno (Leghorn) and many industrial strains of hens, spread all over the world for the white eggs production, derived from the White Leghorn. 

The strongest argument for the conservation of endangered breeds is their value in systems of livestock production and both the production-related data and the genetic data are fundamental [22]. For the Livorno breed, these data are already available [12,23]. The Aim of this study was to present a first contribution on the eggs quality of the endangered Siciliana chicken breed reared in its own environment and to compare the results with the Livorno, an excellent white egg layer, ancestor of the commercial strain worldwide spread, and very appreciated for its ability to adapt itself to extensive organic rearing systems so much that it was proposed as egg layer model for an en-plain air rearing system [24]. So, the objective of this study was to provide additional knowledge on the egg quality of the Livorno breed reared in Sicily to obtain useful data for future conservation programs.

## 2. Material and methods

### 2.1. Animals, Housing, and Feeding

The study was carried out in a commercial poultry farm, located between 36°50’49”20 N and 14°46’29”28 E in the Sicilian region (Italy) that collaborates with the Italian Endangered Breeds Association for the safeguarding of local chicken breeds.

All the birds were reared in the same condition according to organic rearing European legislation, in separated pens exposed to the same protocol, concerning the stocking density, lighting, vaccination, and other rearing procedures. At 20 weeks of age, the 108 laying hens (54 from the Siciliana breed and 54 from the Livorno breed) were divided into six groups of 18 hens each (3 groups for each breed); each group was reared in covered straw-bedded houses (6 bird/m^2^) with free access to pens with the natural grazing (4 m^2^/bird), according to the requirements imposed by the EC Regulation 889/08 [25]. Inside the paddocks, there was a small hut with nests (1 per 6 hens) and perches. Water and layer organic feed was given ad libitum to all groups; more than 90% of ingredients were organically grown, GMO-free and without additives (Table 1). The trial lasted 1 year. During the experimental period, the mean temperature and rainfall was, respectively, 11.1 ± 1 °C and 72.5 ± 26 mm in winter, 14.9 ± 4 °C in spring and 33.6 ± 10 mm, 24.7 ± 2 °C and 7.9 ± 2 mm in summer and 17.2 ± 4 °C and 64.5 ± 15 mm in autumn [26]; indoor and outdoor thermal conditions followed the same trend.

### 2.2. Sample Collection

The production cycle was controlled during the 1st year, from February 2018 to February 2019, and the egg production was daily recorded per group and the average production/head was calculated. Eggs were collected for analyses. A total amount of 360 eggs were gathered, 15 eggs monthly for each breed and 5 for each pen. The eggs (180 per group/year) were stored at 4 °C until analyses (within 2 days).

### 2.3. Physical Analysis 

Whole eggs were individually weighted, length and width (digital sliding calliper, 0.1 mm accuracy) were measured, and the egg shape index was calculated as ((maximum breadth/length) *100) [13]. Then, the breaking strength of the eggshell was measured with the Instron testing machine (model 5542, Instron Ltd., Bucks, UK). A constantly increasing load was applied to the egg lying lengthways until it broke. The applied load at the time of breakage represents the measured strength and the breaking force determined (kgf/mm) [27]. The components of the egg were recovered and the albumen weight, yolk weight, eggshell weight (electronic balance, Mettler PL, Greifensee, Schweiz 0.1 g accuracy), albumen pH (pHmeter InoLAB pH 730P, WTW equipped with an immersion electrode pH Electrode Sentix 81, WTW, Weilheim, Germany) were measured. The percentage of egg components were determined, and the shell index was calculated as the weight of the shell per unit surface area (length × width) and expressed in g/cm^2^ [28]. The yolk color was scored using the Roche egg yolk fan (DSM, Heerlen, Netherlands).

### 2.4. Chemical Composition and Nutritional Quality 

The egg yolk and egg albumen were analyzed to determine the chemical composition according to the ISTISAN method [29] for moisture (1996/34 met. B page 7), ash (1996/34 page 77), lipid (1996/34 met. A page 41), and protein contents (1996/34 page 13). Then, for the chromatographic analysis, lipids from the egg yolk were extracted by the Soxtec system using the petroleum ether as solvent and then were esterified in capped screw top tubes with 2 mL of methanol:sulfuric acid (9:1) [30]. Fatty acid methyl esters (FAMEs) were then cooled at room temperature, and 2 mL of hexane were added. The top layer was removed and placed in chromatography vials and analyzed. The FAMEs were analyzed in a gas chromatograph (Agilent technologies, model 5890, Palo Alto, CA, USA) fitted with Omegawax fused silica capillary column (30m × 0.25 mm i.d. × 0.25 um film thickness: Supelco, Inc., Bellefonte, PA, USA). Helium was the carrier gas, 1 mL/min. The injection volume was 1µL with a split/splitless ratio (80/10). Column parameters were as follows: the initial column temperature was held at 130 °C; increased 4 °C/min to 180 °C (held for 5 min), then increased at 5 °C/min to 230 °C (held for 8 min). [31]. The identification of FAMEs was carried out by comparison to standards. Data were collected automatically, and fatty acids were identified using the computer program Chemstation software (Agilent technologies, Palo Alto, CA, USA). The quantification was carried out by flame ionization detector (F.I.D.) (Agilent technologies, Palo Alto, CA, USA) set at 250 °C. Chromatogram peak areas were acquired and calculated by the Chemstation software (Agilent, technologies, Palo Alto, CA, USA) and expressed in percentage of the identified total fatty acid methyl esters. Each sample of egg yolk was analyzed in triplicate.

The peroxidability index (PI) was calculated according to the equation proposed by Arakawa and Sagai [32]:

(1) PI=(%monoenoic×0.025)+(%dienoic×1)+(%trienoic×2)+(%tetraenoic×4)+(%pentaenoic×6)+(%hexaenoic×8)

The amount of each fatty acid was used to calculate atherogenicity (AI) and thrombogenicity (TI) indices, as proposed by Ulbricht and Southgate [33], and the an hypocholesterolaemic/hypercholesterolaemic ratio (HH) as suggested by Santos-Silva et al. [34]:

(2) $$AI=\frac{C12:0+4\left(C14:0\right)+C16:0}{\Sigma \mathrm{MUFA}+\Sigma n-6\mathrm{PUFA}+\Sigma n-3\mathrm{PUFA}}$$

(3) $$TI=\frac{C14:0+C16:0+C18:0}{\left(0.5\times \Sigma MUFA\right)+\left(0.5\times \Sigma n-6\mathrm{PUFA}\right)+\left(3\times \Sigma n-3\mathrm{PUFA}\right)+\left(\Sigma n-3\mathrm{PUFA}/\Sigma n-6\mathrm{PUFA}\right)}$$

(4) $$HH=\frac{C18:1n-9+C18:2n-6+C20:4n-6+C18:3n-3+C20:5n-3+C22:5n-3+C22:6n-3}{C14:0+C16:0}$$

### 2.5. Statistical Analysis

Data were submitted to statistical analysis by ANOVA using the General Linear Models procedure of SAS (ver. 9.3, 2017) [35]. For physical parameters, data were analyzed with the ANCOVA model and the egg weights were introduced as covariate factor. For the other parameters, the ANOVA model was used, and the fixed effect of genetic type was evaluated. *p*-Values < 0.05 were considered statistically significant. Tukey’s test was applied for post-hoc comparison. 

## 3. Results

Between both groups, hens had a low percentage of eggs laid outside the nest and, consequently, non-marketable eggs (e.g., dirty, cracked or broken). Both strains showed similar values for the hen/day production in the examined productive cycle. In the absence of past selection for these traits, the egg production was 190 egg/head for the Siciliana group and 180 egg/head for the Livorno group. No mortality was observed, and the animal health status was good for the whole period of trial.

### 3.1. Physical Analysis of Eggs 

The average weight (Table 2) was significantly higher (*p* < 0.01) in eggs of the Siciliana hens in comparison with eggs from the Livorno hens. As regards external quality traits (Table 2), the Shape Index was similar for the eggs of both genetic types, while the breaking strength of the Siciliana shell was slightly higher than that observed in the Livorno breed, probably for the higher (*p* < 0.05) shell weight in the Siciliana eggs than that of the Livorno eggs. Also, the Shell Index, the expression of the shell weight per unit of surface, had a certain relation with the breaking strength, showing values slightly higher in the Siciliana eggs than those of the Livorno eggs. As regards internal traits, the albumen (*p* = 0.033) and yolk weights (*p* < 0.0001), and the yolk percentages (*p* = 0.023) were higher in the Siciliana eggs.

The yolk color (Roche scale) showed similar values between groups, much like organic eggs, while lower with respect to commercial eggs.

### 3.2. Proximate Analysis, Fatty Acid Profile and Nutritional Indices

Table 3 shows the chemical composition of different eggs portion.

The moisture (*p* = 0.001) and the ash (*p* = 0.049) showed higher values in the Siciliana egg yolks than those of the Livorno egg yolks. The Siciliana albumens showed higher moisture content (*p* < 0.05) and lower protein and energy content (*p* < 0.0001) than those of the Livorno albumens.

The fatty acid composition of the yolk was similar in the two Italian breeds (Table 4). Egg yolks showed a concentration of the saturated fatty acids (SFAs) (*p* = 0.073), Monounsaturated fatty acids (MUFAs) (*p* = 0.884), and polyunsaturated fatty acids (PUFAs) (0.324) similar in the eggs of both genetic types. Among the fatty acids of nutritional interest, the arachidonic acid (*p* < 0.001) showed a significant lower content in the Siciliana egg yolks than those of the Livorno egg yolks whereas, the linoleic acid, α-Linolenic acid, eicosapentaenoic acid and docosahexaenoic acid showed similar content.

Among the nutritional indices (Table 5), the thrombogenic index was lower in the Siciliana egg yolks then those of Livorno egg yolks (*p* = 0.050), whereas the atherogenic index (*p* = 0.230) and the HH index (*p* = 0.248), showed similar values. The ratios *n*-6/*n*-3 (*p* = 0.437), UFA/SFA (*p* = 0.073) and PUFA/SFA (*p* = 0.193) showed no significant difference between the groups (Table 5).

## 4. Discussion

The Siciliana eggs can be placed in the medium category of marketable eggs, similar to what is evidenced in other autochthonous Italian breeds such as the Modenese (53.73 g), the Romagnola (54.03 g) [13], and the Ermellinata di Rovigo (54.4 g) [36] and slightly lighter than the Ancona (56.7 g) [37] and the Robusta maculate (56.5 g) [36]. Several studies have compared local breeds with commercial strains bred in an organic system and all the commercial layers produced heavier eggs [38,39,40]; however, it is well known that the different genetics of the hybrid and local genotypes is evident in the production performance [36], considering that commercial strains are selected for the high egg productions and egg yields.

The average pH was 8.97 for the Siciliana albumen and 8.95 for Livorno albumen. These values are similar to those reported by Moula et al. [27] for the Famennoise breed (8.86) and by Rizzi et al. [36] in other two Italian breeds, the Ermellinata di Rovigo (9.03) and the Robusta maculate (8.97), but higher than those (8.42–8.44) recorded in the commercial hybrid strain [41]. For the albumen and the yolk, the results confirmed that in hybrid eggs, the albumen percentage is higher, and the yolk percentage was lower than those of Italian eggs [36,42].

The eggshell breaking strength in Siciliana eggs (4.40 kg) was higher than that reported by Sirri et al. [42] in the Romagnola and similar to the commercial hen strain Hy-Line Brown (3.26 versus 4.56 kg, respectively); this result could be related to the higher eggshell weight and percentage observed in the Siciliana. It is well known that the eggshell is the natural packing for the egg contents, and as a result, it is important to obtain a high shell strength, to resist all impacts to which an egg is subjected during the production chain [43]. Broken eggs cause an economic damage in two ways: they cannot be sold as first-quality eggs, and the occurrence of hair cracks raises the risk for bacterial contamination of the broken egg and of other eggs when leaking, creating problems with internal and external quality and food safety [44]. For these reasons, above all in an organic rearing system, the eggshell breaking strength is a very important factor and results from the Siciliana were interesting, similar to the Hy-Line Brown, considering that, generally, the eggshell breaking strength of eggs of similar size is far lower in the white than brown ones, as emerged from the comparison of data reported in the performance objectives guides provided by the breeding companies [42].

The ash content in Siciliana yolk was higher than in Livorno yolk as in other Italian local breed [36]. Rizzi and Marangon (2012) [36] compared Italian strains to commercial Hy-Line hens, reared in an organic system, and they reported a higher ash content in the yolk of Italian local breeds [45] and investigated the effect on the ash content of eggs from organic and conventional-housing systems in brown laying hybrid hens; the authors found that mineral content (mainly P and Zn) of the edible egg portion were 41% and 30% lower in the organic eggs than in the conventional eggs, respectively, and they concluded that probably minerals ingested by hens into organic systems support the metabolism and the immune system rather than being deposited in the egg. Therefore, the high ash percentage in the yolk of Siciliana hens suggest that this local chicken breed fits better to an organic rearing system with more variable environmental conditions with external stressors compared to the stable conditions in the conventional cage barns.

The yolk:albumen ratio was high in the Siciliana like other Italian eggs compared to the Hy-Line White and the Hy-Line Brown commercial strains [36], also reared in an organic system and in accordance with Suk and Park’s [46] observations, where eggs of a dual-purpose pure-breed of the Korean native chicken showed a higher yolk:albumen ratio than that of the commercial egg-type genotype (ISA Brown). The albumen weight is largely related to the egg size; in fact, the genetic improvement lead to a higher albumen weight without the yolk weight reduction permitting to obtain eggs with a higher protein content [47]. In this context, the significant differences in the protein content of albumens observed in this study where the laying hens, the Siciliana and the Livorno, have the identical rearing system and age, could be traceable to the genetic specificity [48]. The yolk composition showed a lipid content slightly lower in Siciliana eggs than Livorno eggs (*p*= 0.357), and a similar content with commercial eggs [49]. Some authors have stated that an inverse relationship exists between the egg yolk cholesterol content and the yolk size [50], so the higher yolk size and percentage of Siciliana eggs compared to Livorno eggs, might suggest a lower cholesterol content and a more healthier lipids profile, according to Gilbert et al. (2000) [51] who observed more healthier and valuable eggs when the cholesterol content is reduced.

Eggs have been considered as a principle food item for the human consumption over the history as they provide most of the nutritional component, as suggested by the Recommended Daily Allowance (RDA) [52]. It is well known a hens’ diet strongly influences the egg composition [53,54,55,56,57]. The unsaturated fatty acids play a prominent role as total plasma and low-density lipoprotein (LDL) cholesterol reducers [49], and for this reason, several studies had been conducted to raise the unsaturated fatty acid content in eggs by using dietary fat sources, such as the natural oil containing PUFA [58]. Attia et al. (2015) [57] performed a study in Saudi Arabia to monitor the fatty acids, cholesterol profiles, atherogenic and thrombogenic indices of eggs from commercial layers, collected in the retail market, and their capability to RDA. Authors showed an UFA content range from 58.54% to 62.53%, lower than that in Livorno eggs (64.31%), which was in turn lower than that in Siciliana eggs (65.55%). These results, considering that the commercial layers are selected and produced under highly controlled conditions and accurate feeding strategies, confirmed that the breed and strain of layers [49,59,60] affect the egg quality and composition and the genotypes show different abilities to incorporate fatty acids despite the poorer living conditions in an organic system (less controlled environment and less equilibrated rations) [61].

Consumers are progressively more interested in the food and food components that provide health benefits and prevention of diseases. Accordingly, nutritional indices were introduced [33]. Atherogenic and thrombogenic indices can be considered markers of the fat quality [62], and a healthy diet is characterized by low AI and TI [52,63,64], because the fatty acid profile can promote or reduce the atherosclerosis and coronary thrombosis [41]. Moreover, eggs’ fatty acids and cholesterols are important components from the health and consumption prospective for humans particularly in terms of PUFA and ω-3 fatty acids [57,65]. From this point of view, eggs with a higher UFA/SFA ratio and low AI and TI are recommended for a healthy diet [52,66,67]. The fatty acid profile from Siciliana eggs and Livorno eggs affected the nutritional indices derived from their proportion. In this study, the atherogenic and thrombogenic indices in both groups were optimal from a nutritional point of view and were much lower that the Gonzalez-Munoz’ et al. [68] results in commercial improved layers (Hy-Line and Warren) fed different dietary fats and the Attia et al. (2015) [57], results in eggs from market retails whereas, they are similar to Mugnai’ et al. [31] results in hens rearing in an organic condition. Specifically, Siciliana eggs showed a lower thrombogenic index than that of Livorno eggs and a similar atherogenic index. Probably, the significant lower arachidonic acid content observed in Siciliana egg yolk lead to a lower thrombogenic index and could be considered to be thrombogenic. Moreover, the arachidonic acid (C20:4n6) is an important precursor in eicosanoids’ synthesis and through the action of the cyclooxygenase and peroxidase enzymes gives rise to prostaglandin PGH2 (C_20_H_32_O_5_), which in turn is transformed into various substances including prostaglandins involved in inflammatory processes [69]. Although, recent studies have indicated that eggs are not a predisposing factor for the risk of cardiovascular pathologies [70,71,72], the lower value of AI and TI observed suggested a better fat quality for eggs produced by Siciliana hens. From a nutritional point of view, in addition to the evaluation of AI and TI, a good approach to the nutritional evaluation of fat should be the application of another index based on healthy effects of fatty acids, the ratio hypocholesterolaemic:hypercholesterolaemic fatty acids (HH), calculated according to present knowledge of the effects of individual fatty acids on cholesterol metabolism [62,73,74]. The similar values observed for the HH index of Siciliana and Livorno eggs, confirmed the optimal nutritional value of eggs from the local chicken breed. From a nutritional health point of view, the optimal indices in Siciliana eggs (AI, TI, PI, HH), similar to those found by Mugnai et al. [31] in Ancona’ hens reared in an organic condition with the grass intake for improve eggs quality, could play an important role 

The primary aim of the organic production system is optimizing ecological productions that promotes the biodiversity, environmental sustainability and food safety. Fast-growing animals are not adapted to an organic system and the health and welfare problems are recurrent (Network for Animal Health and Welfare in Organic Agriculture, 2002) [24,75]. Leenstra et al. (2012) [76] carried out a study on the performance of commercial laying hen genotypes in a free range and organic farms in Switzerland, France, and The Netherlands; they showed that the overall effect of the breeding system (organic versus conventional free range) on the egg production and mortality was significant, with a higher mortality and a lower egg production in organic hens. Siciliana and Livorno hens are a low growing local breed with a poor productive performance. The egg number and size are not a benefit for farmers, but their egg quality, mainly for the Siciliana, demonstrated an optimum adaptation to variables conditions. The capability of the native breed genotypes either to synthesize or to transfer to tissue a high quantity of some fatty acids, minerals, or other molecules considered as functional factors for the human health could be an advantageous factor for rearing these genotypes in an organic farming or in a free-range system condition [61], and in its native environment, Siciliana hens showed an optimum nutritional profile of eggs, compared to the Livorno, appreciated as excellent white eggs layers more adapted to an organic rearing system.

Conservation of endangered native breeds involves the genetics, social, cultural and heritage factors, and local breeds are an integral part of the evolving diversity of a region. The FAO Global Strategy for the Management of Farm Animal Genetic Resources (FAnGR), complying with Convention on Biological Diversity (CBD), recommend the increase of the rearing and diffusion of purebreds or local genotypes [77]. Considering that, for the conservation of endangered breeds, the strongest argument is their value in systems of the livestock production, our results can be considered a first contribute to identify the performance and nutritional characterization of eggs of Siciliana hen, because the market place changes continually and a breed that could not compete in the past may be a strong competitor in the future.

## 5. Conclusions

The data showed similar values for the physical–chemical characteristics, fatty acid profile, and nutritional indices of Siciliana and Livorno eggs highlighting numerous valuable quality traits of eggs from Siciliana and Livorno breeds which might be taken into account for the safeguarding and utilization of this currently low exploited Italian chicken. Nowadays, a huge number of local European breeds suffer a critical reduction in number, due mainly to the agriculture industrialization and introduction of the productive and improved breeds, but, in recent years, the interest for the safeguarding biodiversity, animal welfare and healthy nutritional quality of food has been increasing. The FAO Global Strategy recommends the increase of the rearing and diffusion of purebreds or local genotypes, therefore, the results of this study can be considered a first contribute to identify the performance and nutritional characterization of eggs of Siciliana hen and to encourage the farmers to breeding native breeds particularly suitable of an organic system. Changes in the consumer demand could convince breeders to safeguard this endangered breed, promoting this egg as a food that contributes to the preservation of biodiversity, animal welfare, and maintenance of the typical Sicilian environment.

## Figures and Tables

**Table 1:** Chemical composition of the diet. *

Nutrients **	% DM ***
Crude Protein	16.50
Ether Extract	4.50
Cellulose	5.00
Ash	13.20
Calcium	3.90
Sodium	0.16
Phosphorus	0.60
Methionine	0.35
Lysine	0.80

* Ingredients of the diet: ground maize, sorghum, spelt, rye, dehydrated alfalfa flour, carob, soybean cake, sunflower cake, filed bean, wheat, protein pea, wheat bran, calcium carbonate, flaxseed, sodium chloride. All the ingredients were GMO free. Diet did not contain additives. ** According to (EC) No 889/2008. *** DM: Dry Matter.

**Table 2:** Effect of genetic type on physical characteristics of eggs.

Trait	Breed		SEM **	*p*
	Siciliana	Livorno		
Egg weight (g)	54.93	48.16	0.150	0.0002
Shell weight (g)	7.52	6.51	0.181	0.041
Albumen weight (g)	29.54	26.67	0.179	0.033
Yolk weight (g)	17.76	14.64	0.142	< 0.0001
Shell (%)	13.70	13.49	0.196	0.893
Albumen (%)	53.76	55.37	0.193	0.408
Yolk (%)	32.35	30.45	0.177	0.023
Shape Index (%)	71.07	73.09	0.189	0.164
Breaking strength (kgf/mm) *	4.40	3.83	0.194	0.458
Shell index (g/cm^2^)	0.31	0.29	0.190	0.205
Albumen pH	8.96	8.95	0.377	0.837
Yolk color	9.49	9.64	0.196	0.886

* Breaking force applied to measure the strength of uncracked eggs. ** SEM: standard error of the mean.

**Table 3:** Chemical composition of yolk and albumen.

Parameters	Breed		SEM	*p*
	Siciliana	Livorno		
Yolk				
Moisture (%)	49.59	48.76	0.162	0.001
Crude Protein (%)	16.75	17.45	0.354	0.350
Lipid (%)	30.49	31.50	0.354	0.357
Ash (%)	2.34	1.90	0.281	0.049
Energy (kJ/100 g)	1412.83	1462.15	0.356	0.382
Albumen				
Moisture (%)	88.73	87.83	0.219	0.008
Crude Protein (%)	9.93	11.58	0.135	0.0001
Ash (%)	1.11	1.28	0.282	0.050
Energy (kJ/100 g)	170.63	196.90	0.145	0.0001

**Table 4:** Fatty acid composition of eggs yolk (g·100 g^−1^ FAME) *.

Items	Breed		SEM	*p*
	Siciliana	Livorno		
Myristic acid (C14:0)	0.33	0.34	0.330	0.676
Palmitic acid (C16:0)	25.38	26.06	0.315	0.330
Palmitoleic acid (C16:1n7)	3.00	3.18	0.327	0.567
Stearic acid (C18:0)	8.68	9.22	0.299	0.170
Oleic acid (C18:1n9)	45.83	45.42	0.331	0.751
Vaccenic acid (C18:1n7)	1.93	1.96	0.331	0.742
Linoleic acid (C18:2n6)	11.88	10.68	0.305	0.222
α-Linolenic acid (C18:3n3)	0.36	0.28	0.301	0.191
Arachidic acid (C20:0)	0.03	0.03	0.314	0.319
Eicosenoic acid (C20:1n9)	0.24	0.22	0.316	0.342
Arachidonic acid (C20:4n6)	1.64	1.94	0.183	0.001
Behenic acid (C22:0)	0.03	0.04	0.298	0.168
Eicosapentaenoic acid (C22:5n3)	0.11	0.14	0.311	0.274
Docosahexaenoic acid (C22:6n3)	0.56	0.49	0.298	0.168
SFAs	34.45	35.69	0.276	0.073
MUFAs	51.00	50.78	0.333	0.844
PUFAs	14.55	13.53	0.315	0.324

* The concentration of fatty acids was expressed as g·100 g^−1^, considering 100 g the sum of the areas of all identified fatty acid methyl esters (FAMEs). SFA: Sum of the saturated fatty acids. MUFA: Sum of the monounsaturated fatty acids. PUFA: Sum of the polyunsaturated fatty acids.

**Table 5:** Nutritional indices and ratios of egg yolk.

Items	Breed		SEM	*p*
	Siciliana	Livorno		
AI	0.41	0.43	0.306	0.230
TI	0.97	1.03	0.266	0.050
PI	25.57	25.02	0.329	0.651
HH	2.36	2.24	0.308	0.248
Σn-3	1.03	0.91	0.293	0.139
Σn-6	13.52	12.62	0.317	0.356
n-6/n-3	13.21	13.87	0.322	0.437
UFAs/SFAs	1.90	1.80	0.276	0.073
PUFAs/SFAs	0.42	0.38	0.302	0.193

AI: atherogenic index; TI: thrombogenic index; PI: peroxidability index; HH: hypocholesterolaemic/hypercholesterolaemic ratio; Σn-3: Sum of the polyunsaturated fatty acids of n-3 series; Σn-6: Sum of the polyunsaturated fatty acids of n-6 series.
